# BERTopic_Teen: a multi-module optimization approach for short text topic modeling in adolescent health

**DOI:** 10.3389/fpubh.2025.1608241

**Published:** 2025-08-12

**Authors:** Yiqiang Feng, Ziao Chen, Yuxin Zhang, Wenyuan Huang, Xuanming Zhang, Siyu He

**Affiliations:** ^1^School of Marxism, Sichuan Agricultural University, Chengdu, China; ^2^College of Law, Sichuan Agricultural University, Yaan, China; ^3^College of Information Engineering, Sichuan Agricultural University, Yaan, China; ^4^College of Mechanical and Electrical Engineering, Hohai University, Changzhou, China

**Keywords:** adolescent health, social media analytics, topic modeling, BERTopic, health systems

## Abstract

Adolescent health has become a critical dimension in the digital era, as social media platforms emerge as vital sources of real-time behavioral data for informing sustainable and equitable public health strategies. However, conventional topic modeling methods often struggle with the semantic sparsity and noise inherent in short-form texts. The study proposes BERTopic_Teen, an enhanced topic modeling framework optimized for adolescent health-related tweets. The model incorporates three key innovations: a Popularity Deviation Regularizer (PDR) to suppress high-frequency generic terms and amplify domain-specific vocabulary; a Dynamic Document Embedding Optimizer (DDEO) that adaptively selects optimal UMAP dimensions based on silhouette scores; and a Probabilistic Reassignment Matrix (PRM) to reassign outlier documents to relevant topic clusters. Using a dataset of 64,441 tweets (61,039 successfully classified), experimental results show that BERTopic_Teen outperforms LDA, NMF, Top2Vec, and the original BERTopic in all key evaluation metrics. It achieves a 16.1% improvement in topic coherence (NPMI = 0.2184), higher topic diversity (TD = 0.9935), and lower perplexity (1.7214), indicating superior semantic clarity, topic distinctiveness, and modeling stability. These findings suggest that BERTopic_Teen offers a robust solution for extracting meaningful topics from social media data and advancing public health surveillance.

## 1 Introduction

Adolescent health is an important indicator of a country's and society's overall development, and the dynamic interactions between their physiological, psychological, and behavioral characteristics directly impact the effectiveness of public health systems (1). According to the United Nations population statistics, the global youth population aged 15–24 has reached 1.2 billion, and it is projected to increase to 1.3 billion by 2030 (2). In the context of digitization and globalization, adolescent health faces multifaceted challenges: increased social media usage leading to disrupted sleep cycles (an average reduction of 0.8 h per day) (3), changes in dietary patterns contributing to an increased risk of metabolic syndrome (OR = 1.32) (4), and a significant rise in the detection of anxiety symptoms during the COVID-19 pandemic (from 12.9 to 25.6%) (5). These phenomena urgently require data analysis to reveal their spatiotemporal evolution patterns, providing a scientific basis for the formulation of long-term, sustainable public health policies.

With the advent of the digital age, the unprecedented availability of massive data and the concurrent advancement in computational capabilities have fundamentally reshaped the paradigms of social science research (6). The aggregation of large-scale data captures the digital footprints of individuals and collectives, offering new opportunities to uncover patterns in human behavior (7). Among these sources, social media platforms have emerged as indispensable tools, particularly in research on adolescent health, psychological wellbeing, and behavioral trends, as they provide real-time, large-scale, and behaviorally rich datasets. Twitter alone generates ~500 million tweets daily, with a significant portion involving health-related content (8, 9). Compared to traditional epidemiological surveys with response rates around 58% (10), social media data enable high-frequency updates and real-time monitoring of collective dynamics (11). However, their unstructured nature, especially the dominance of short texts (over 70%), poses significant challenges for topic modeling (12). BERTopic (13), which leverages semantic embeddings from pre-trained language models along with Uniform Manifold Approximation and Projection (UMAP) for dimensionality reduction, is compatible with multiple clustering algorithms, such as HDBSCAN, and offers a flexible framework for extracting evolving topics from such data. However, it faces three key limitations in health-related applications: (1) fixed UMAP dimensions may limit its ability to capture the full complexity of the data (14); (2) high-frequency general terms (e.g., “health,” “teen”) compromise topic specificity (15); and (3) HDBSCAN discards outlier data during the clustering process (16), potentially missing critical early signals of emerging health events.

Although big data analytics, particularly in the areas of machine learning and natural language processing (NLP), holds significant potential for applications in the social sciences, current discussions remain largely focused on theoretical critique and conceptual exploration, with relatively little emphasis on practical implementation. While big data methods can uncover novel patterns in social phenomena, effectively interpreting these patterns and translating them into actionable research outcomes continues to pose a major challenge for the academic community. As NLP technologies rapidly advance, the emergence of new modeling algorithms has further increased the complexity of analytical processes. At the same time, these developments have introduced new strategies for selecting and applying diverse analytical approaches. Consequently, in domains such as adolescent health and social media research, a pressing question emerges: how can these advanced techniques be leveraged to accurately capture health-related discourse on social media and reveal its dynamic patterns of change?

To enhance the precision of health-related topic analysis, this study proposes BERTopic_Teen, an improved version of the BERTopic framework, incorporating the following three computational optimizations:

(1) Popularity Deviation Regularizer (PDR). To suppress high-frequency generic terms, we apply an exponential decay based on term rank, reducing their weights while highlighting domain-specific vocabulary.
(2) Dynamic Document Embedding Optimizer (DDEO). Adapts the dimensionality of UMAP based on the maximization of the silhouette score, selecting an optimal dimension between two and 40.
(3) Probabilistic Reassignment Matrix (PRM). Reallocates outlier documents to their nearest topic clusters using cosine similarity, with a threshold of *P* > 0.15.

Empirical analysis based on 64,441 adolescent health-related tweets (of which 61,039 were successfully classified) demonstrates that the improved BERTopic_Teen model outperforms the original BERTopic across all evaluation metrics. Specifically, it achieves a 16.1% improvement in topic coherence (NPMI = 0.2184 vs. 0.1882), higher topic diversity (TD = 0.9935), indicating more distinct and non-overlapping topics, and a significantly lower perplexity (1.7214 vs. 2.0580), suggesting enhanced model stability and better overall fit.

## 2 Literature review

### 2.1 Social media analysis of health topics

Social media data have emerged as a vital resource for public health research, offering high temporal resolution and the ability to reflect collective behavior in real time, thereby providing a valuable complement to traditional epidemiological approaches (17, 18). By analyzing text content from platforms such as Twitter and Facebook, researchers can track the dissemination of health topics, fluctuations in public sentiment, and the immediate effects of policy interventions (19).

Compared to conventional data sources like surveys, clinical records, and government statistics, social media offer shorter data collection cycles and broader population coverage. This is particularly advantageous for adolescent populations, where privacy concerns and low participation rates often result in survey response rates below 58% (20), making it difficult to capture sensitive behaviors such as internet addiction or disordered eating. Moreover, public health emergencies like the COVID-19 pandemic have highlighted the limitations of traditional monitoring systems in terms of response speed and real-time adaptability (21).

Twitter alone produces ~500 million tweets per day, with 7.3% related to health content. Rich metadata, such as timestamps and geolocation, make these data suitable for analyzing spatiotemporal health trends. Previous studies have demonstrated the potential of social media in early warning systems; for example, Hswen et al. (22) detected a vaping-related lung disease outbreak in 2021 3 weeks earlier than traditional surveillance systems. Social media data have also been used to track public attitudes toward health policies, such as the geographic diffusion of vaccine acceptance (23).

Health-related tweets often exhibit informal characteristics, such as abbreviations, slang, and emojis, which pose challenges for natural language processing (NLP) techniques (24). Additionally, prior studies have shown that tweets are typically concise, with limited word counts compared to formal text sources (25), which can exacerbate semantic sparsity and reduce topic modeling accuracy. Deep learning approaches have partially mitigated these issues. For instance, BERTopic reduces the number of irrelevant topic words compared to traditional models by leveraging contextual semantic embeddings, which improve the semantic coherence of generated topics (26), and Gaur et al. (27) applied attention mechanisms to achieve 89% topic relevance in mental health tweet analysis. However, most existing methods still struggle with filtering high-frequency noise terms and identifying long-tail domain-specific terms, lacking effective strategies to balance the two.

### 2.2 Advances in topic modeling techniques

Topic modeling has evolved significantly in recent years, with a methodological transition from early statistical approaches to deep learning-based techniques. The core objective remains the same: uncovering latent semantic structures in text through unsupervised learning. Based on their underlying methodologies, existing topic modeling approaches can be broadly categorized into probabilistic models, matrix factorization methods, and neural or embedding-based techniques. these categories exhibit distinct strengths in terms of semantic representation, interpretability, and computational efficiency.

Traditional topic modeling was initially dominated by early statistical approaches, such as Latent Dirichlet Allocation (LDA) (28) and Non-negative Matrix Factorization (NMF) (29). LDA models a three-layer probabilistic structure, including document, topic, and word, assuming that each document is generated from a mixture of latent topics, each represented by a distribution over words. In contrast, NMF factorizes the document-word matrix into two non-negative matrices representing document-topic and topic-word relationships. Although these methods perform robustly on long-form texts, they rely on the “bag-of-words” assumption, neglect contextual semantics and requiring the number of topics to be predefined (30).

With the rise of deep learning, Neural Topic Models (NTMs) have attracted increasing attention. Tu et al. (31) were among the first to incorporate Variational Autoencoders (VAEs) into topic modeling, proposing an end-to-end framework to learn latent topic distributions. Dieng et al. (32) extended this line of research by introducing Embedding Topic Models (ETM), which integrate word embeddings to enhance topic coherence and interpretability, but at the cost of higher computational complexity and longer training times due to their reliance on neural variational inference. They continue to rely on manually preset topic numbers, which limits their applicability in dynamic health-related topic detection.

More recently, topic modeling methods that combine pre-trained language models with clustering algorithms have achieved notable progress. A representative example is BERTopic, which employs a four-stage process for efficient topic extraction by generating contextualized document embeddings using models such as BERT, applying UMAP for dimensionality reduction, and clustering documents using HDBSCAN (a density-based method selected in this study), followed by extracting topic keywords via class-based TF-IDF (c-TF-IDF) weighting.

### 2.3 Contributions of this study

As summarized from prior analysis (14–16), this study addresses three main limitations of the original BERTopic model by introducing the following modular enhancements:

(1) Popularity Deviation Regularizer (PDR). Applies exponential decay to penalize high-frequency generic terms and upweights domain-specific vocabulary to improve topic distinctiveness.
(2) Dynamic Document Embedding Optimizer (DDEO). Selects optimal UMAP dimensions based on silhouette scores to minimize semantic information loss during dimensionality reduction.
(3) Probabilistic Reassignment Matrix (PRM). Reassigns HDBSCAN-identified outliers to the most semantically similar topic clusters using a soft clustering approach.

## 3 Data and methods

### 3.1 Data collection and preprocessing

The study collected tweets related to “adolescent health” using the official Twitter API v2. The retrieval keywords are listed in Table 1. The data spans the period from January 1, 2018, to December 31, 2024, resulting in a total of 64,441 original tweets.

**Table 1:** Keyword list for adolescent health topic.

**Keywords**
“Teen health,” “adolescent mental health,” “youth health education,” “adolescent health”

The data preprocessing procedure involved the following steps:

Stopword Removal. Common high-frequency terms unrelated to semantic content, such as “and” and “the,” were removed using the default English stopword dictionary provided by the NLTK library (33).


(1) $$\begin{array}{l} T^{\prime }=T\backslash \left\{w|w\in StopWords\right\} \end{array}$$

where *T* represents the original word set of the tweet, and *T*′ denotes the set after stopword removal.

Deduplication. To eliminate potential duplicate tweets, we used hash matching and cosine similarity between sentence embeddings generated via the Sentence-BERT model from the sentence-transformers library.


(2) $$\begin{array}{l} Sim\left(t_{i},t_{j}\;\right)<\epsilon \Rightarrow retain\;t_{i} \end{array}$$

where Sim() denotes the similarity function between two texts, and ϵ is the similarity threshold.

Emoji and URL Filtering. Regular expressions (Regex) were used to detect and remove noisy content such as emojis and hyperlinks. Unicode ranges corresponding to emojis were replaced with empty characters, and strings beginning with “http(s)://” or “www” were identified and removed as external links. As shown in Table 2, an example of tweet content before and after preprocessing is provided.

**Table 2:** Example of tweet content before and after preprocessing.

**Type**	**Tweet content**
Original	@Aaron_GDAC Excited for you! I'll be working on more portraits and training as a child and adolescent mental health coach!
After preprocessing	aaron_gdac excited working portrait training child amp adolescent mental health coach

### 3.2 BERTopic model

BERTopic is a topic modeling method based on the pre-trained language model BERT (Bidirectional Encoder Representations from Transformers), designed to identify and analyze latent topics within large-scale text corpora. Its core workflow consists of four stages.

#### 3.2.1 Text embedding

The model uses the paraphrase-multilingual-MiniLM-L12-v2 embedding model to convert textual input into embedding vectors (34). An embedding vector refers to a dense, low-dimensional representation of input data, such as text, images, or categorical features, generated by an embedding function that maps complex, high-dimensional, or sparse inputs into a continuous vector space. This transformation produces high-dimensional semantic representations, which are then reduced using UMAP to mitigate the “curse of dimensionality” and improve clustering efficiency.

Specifically, let a text *T* = {*w*_1_, *w*_2_, …, *w*_*n*_} consist of *n* tokens, where each token *w*_*i*_ is associated with a BERT embedding vector *v*_*i*_. The overall embedding representation of the text, denoted as *v*_*T*_, is calculated as the average of all token embeddings:


(3) $$\begin{array}{l} v_{T}=\frac{1}{n}\overset{n}{\underset{i=1}{\sum }}v_{i} \end{array}$$

where *v*_*T*_ represents the embedding of text *T*, and *v*_*i*_ is the BERT embedding vector of token *w*_*i*_.

#### 3.2.2 Text dimensionality reduction

This study employs the UMAP algorithm to reduce the dimensionality of BERT-derived embeddings. UMAP can preserve both local and global structures in high-dimensional data:


(4) $${\text{v}}_{T}^{'}=\text{UMAP}(v_{T})$$

Where ${\text{v}}_{T}^{'}$ denotes the low-dimensional embedding vector of the input text after dimensionality reduction.

#### 3.2.3 Text clustering

To cluster semantically similar documents, we adopted the HDBSCAN (Hierarchical Density-Based Spatial Clustering of Applications with Noise) algorithm, which is particularly effective for high-dimensional, sparse, and noisy data such as short-form tweets. HDBSCAN constructs a hierarchical clustering based on density estimation and selects the most stable clusters, offering two key advantages over traditional algorithms like K-Means: it does not require a predefined number of clusters, and it robustly handles outliers by assigning low-density points to noise.

The clustering process includes the following steps:

(1) Mutual Reachability Distance. For any two points *x*_*i*_ and *x*_*j*_, their mutual reachability distance is defined as:

(5) $$\begin{array}{l} {\text{d}}_{\text{mreach}}\left({\text{x}}_{\text{i}},{\text{x}}_{\text{j}}\right)=\text{max}\left(\text{core}\left({\text{x}}_{\text{i}}\right),\text{core}\left({\text{x}}_{\text{j}}\right),\text{d}\left({\text{x}}_{\text{i}},{\text{x}}_{\text{j}}\right)\right) \end{array}$$

Where *d*(*x*_*i*_, *x*_*j*_) is the Euclidean distance between the two points, and *core*(*x*_*i*_) is the distance from *x*_*i*_ to its farthest neighbor among the *k* nearest neighbors, with *k* defined by the MinSamples parameter.

(2) Graph Construction and Clustering. A weighted graph is built where edge weights equal the mutual reachability distances. HDBSCAN then constructs a minimum spanning tree and derives a hierarchical clustering. Cluster stability *S*(*C*) is computed as:

(6) $$\begin{array}{l} S\left(C\right)=\underset{\left(i,j\right)\in C}{\sum }\left({\text{λ}}_{birth}\left(C\right)-{\text{λ}}_{death}\left(C\right)\right) \end{array}$$

Where λ_*birth*_(*C*) and λ_*death*_(*C*) represent the threshold distances at which the cluster emerges and dissolves.

(3) Outlier Detection. Data points that fail to meet minimum density criteria are labeled as noise. Outlier likelihood is estimated based on core distance:

(7) $$\begin{array}{l} \text{OutlierScore}\left(x_{i}\right)=\frac{\text{Core Distance}\left(x_{i}\right)}{\text{Mean Core Distance of Cluster}} \end{array}$$

A higher score indicates a higher probability of being an outlier.

(4) Final Cluster Assignment. Points are assigned to clusters {*C*_1_, *C*_2_, …, *C*_*k*_}, or to a noise cluster *C*_*noise*_, according to:

(8) $$\text{ClusterLabel}\left(x_{i}\right)=\{\begin{array}{cc} C_{k} & if\;x_{i}\in C_{k}\\ -1 & if\;x_{i}\in C_{\text{noise}} \end{array}$$

#### 3.2.4 Topic representation

To extract representative keywords for each topic, we adopted class-based TF-IDF (c-TF-IDF), which highlights terms that are frequent within a topic but rare across others. The relevance of each word *w*_*i*_ in topic *t*_*j*_ is calculated by:


(9) $$\begin{array}{l} c-TF-IDF\left(w_{i},t_{j}\right)=\frac{TF\left(w_{i},t_{j}\right)}{\sum_{i}TF\left(w_{i},t_{j}\right)}\times \log\frac{N}{\sum_{d\in D}I\left(w_{i},d\right)} \end{array}$$

In addition, to extract the most representative topic keywords, the Maximal Marginal Relevance (MMR) method is applied to promote term diversity and avoid redundancy:


(10) $$\begin{array}{l} \text{MMR}\left(w_{i}\right)=\text{λ}\cdot \text{Sim}\left(w_{i},q\right)-\left(1-\text{λ}\right)\cdot \underset{w_{j}\in S}{\max}\text{Sim}\left(w_{i},w_{j}\right) \end{array}$$

**Figure 1:**
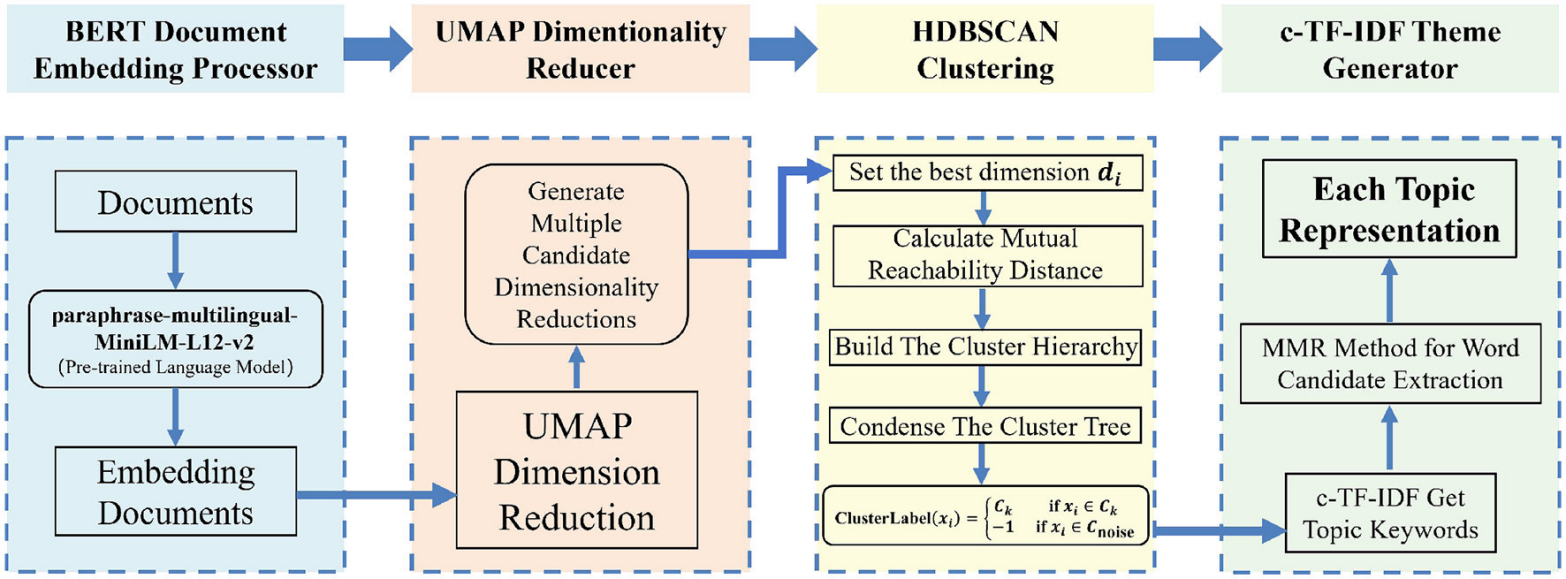
The architecture of the BERTopic framework.

The structure of the BERTopic model is shown in Figure 1.

### 3.3 Improved BERTopic framework

#### 3.3.1 Popularity deviation regularizer (PDR)

High-frequency generic terms in health-related tweets (e.g., “health,” “teen”) often lead to topic homogeneity. To mitigate this issue, the Popularity Deviation Regularizer (PDR) employs a twofold mechanism:

(1) Exponential Decay Weighting. An exponential penalty is applied to the top 10% of high-frequency terms based on their frequency rank:

(11) $$\begin{array}{l} w\left(t\right)=\text{TF}-\text{IDF}\left(t\right)\times e^{-\alpha \cdot \text{rank}\left(t\right)} \end{array}$$

Where α = 0.05, and *rank*(*t*) denotes the frequency rank of term *t* (with the most frequent term ranked as 1).

(2) Domain Dictionary Enhancement. Terms included in the adolescent health vocabulary (see Table 3), such as “bullying” and “disorder,” are upweighted by a factor of 1.5 to emphasize domain-specific semantics.

**Table 3:** The adolescent health vocabulary.

**Keywords**
Sleep, stress, depression, anxiety, nutrition, bullying, self-harm, substance, screen, cyber, mental health, wellbeing, addiction, mindfulness, exercise, suicide, therapy, meditation, resilience, counseling, psychology, social media, selfcare, trauma, emotion, diagnosis, insomnia, disorder, coping, psychotherapy

#### 3.3.2 Dynamic document embedding optimizer (DDEO)

To address the instability in semantic information retention during the UMAP dimensionality reduction process, this study introduces the DDEO. The goal is to adaptively determine the optimal UMAP dimensionality to improve topic modeling quality. The DDEO process involves the following steps:

(1) Dimension Range Setting. The UMAP output dimension *d* is predefined within the range *d*∈[2, , 40], which covers typical semantic representation requirements for textual embeddings.
(2) Silhouette Score Evaluation. For each candidate dimension *d*, the silhouette score *S*(*d*) is calculated based on the clustering result:

(12) $$\begin{array}{l} S\left(d\right)=\frac{1}{N}\overset{N}{\underset{i=1}{\sum }}\frac{b\left(x_{i}\right)-a\left(x_{i}\right)}{\max\left\{a\left(x_{i}\right),b\left(x_{i}\right)\right\}} \end{array}$$

where *a*(*x*_*i*_) is the average intra-cluster distance for sample *x*_*i*_, and *b*(*x*_*i*_) is the smallest average distance from *x*_*i*_ to any other cluster.

(3) Optimal Dimension Selection. The dimension that maximizes the silhouette score is selected as the optimal UMAP parameter.

#### 3.3.3 Probabilistic reassignment matrix (PRM)

Let *T* = {*t*_1_, *t*_2_, ..., *t*_*M*_} be the set of *M* topic clusters, and *N* be the total number of documents. For any document *d*_*i*_, the probability *P*(*d*_*i*_, *t*_*j*_) represents the likelihood of *d*_*j*_ belonging to topic *t*_*j*_, calculated based on the semantic relationship between the document and the topic. To handle outliers discarded by HDBSCAN, the PRM reallocates them using soft clustering via semantic similarity:

(1) Document–Topic Probability Matrix Construction. Each document *d*_*i*_ is associated with a probability distribution over topics:

(13) $$\begin{array}{l} P\left(d_{i}\right)=\left[P\left(d_{i},t_{1}\right),P\left(d_{i},t_{2}\right),\ldots ,P\left(d_{i},t_{M}\right)\right] \end{array}$$

Where $\overset{M}{\underset{j=1}{\sum }}P\left(d_{i},t_{j}\right)=1$, indicating the distribution of the document across all topics.

(2) Topic Probability Estimation. For each document, the conditional probability of belonging to topic *t*_*j*_ is estimated using a softmax function over the mutual reachability distances:

(14) $$\begin{array}{l} P\left(t_{j}|d_{i}\right)=\frac{\exp\left(-d_{mreach}\left(d_{i},t_{j}\right)\right)}{\sum_{k=1}^{M}\exp\left(-d_{mreach}\left(d_{i},t_{k}\right)\right)} \end{array}$$

Where *d*_*mreach*_(*d*_*i*_, *t*_*j*_) denotes the semantic distance between document *d*_*i*_ and topic *t*_*j*_. This ensures the resulting probabilities sum to 1.

(3) Topic Assignment Update. After constructing the probability matrix, documents, particularly outliers, are no longer hard-assigned to a single cluster. Instead, each document is associated with a full probability distribution across all topics, enhancing topic coverage and representation.

**Figure 2:**
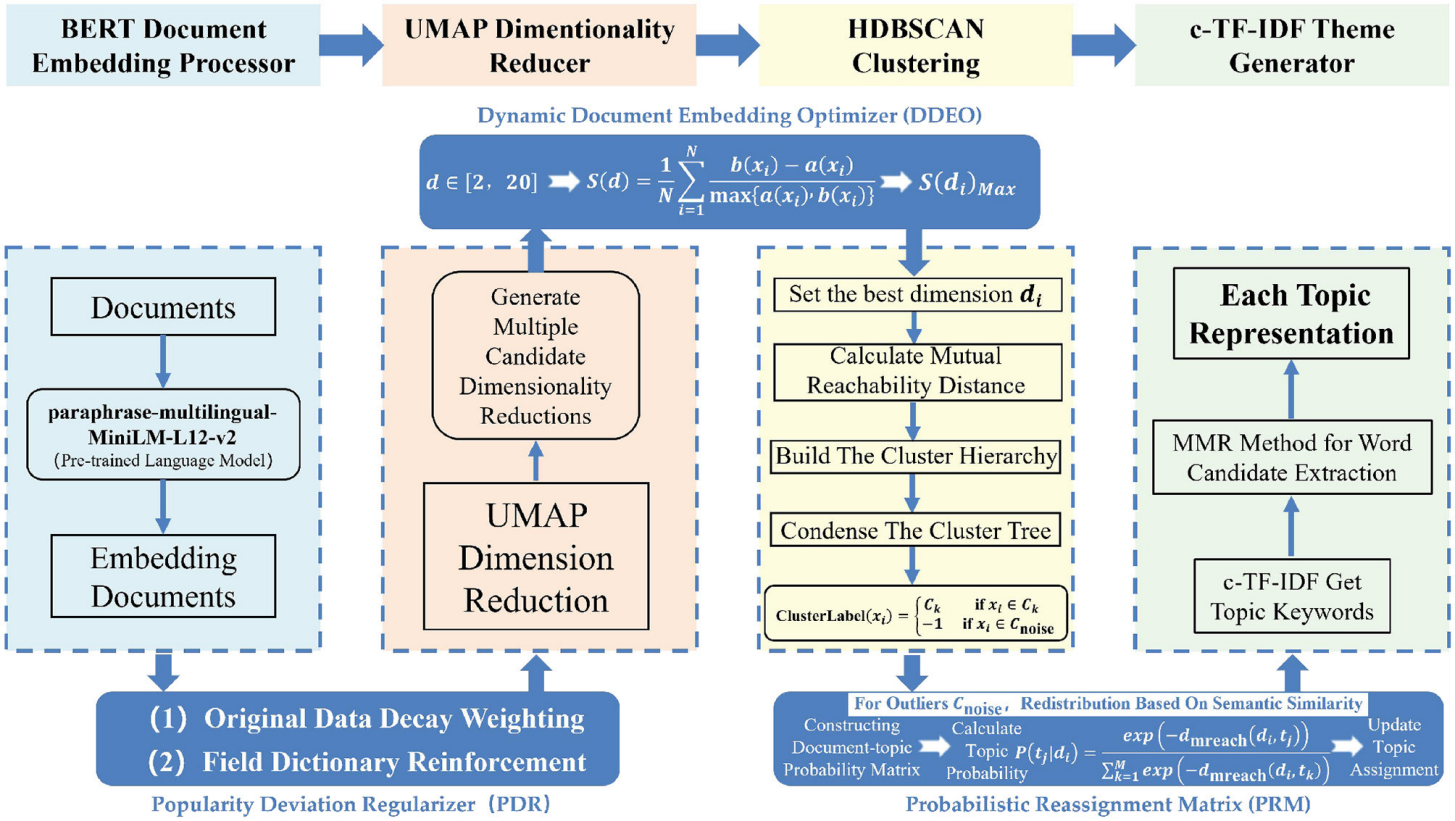
The architecture of the BERTopic_Teen framework.

The structure of the BERTopic_Teen model (BERTopic with PDR, DDEO, and PRM modules) is shown in Figure 2.

### 3.4 Comparative experiment design

#### 3.4.1 Baseline models

The study compares the proposed model with four representative topic modeling approaches.

(1) Latent Dirichlet Allocation (LDA). A probabilistic generative model that assumes each document is generated from a mixture of latent topics. The number of topics must be predefined. The generative process is represented as:

(15) $$\begin{array}{l} P\left(w|\theta ,\phi \right)=\overset{D}{\underset{d=1}{\prod }}\overset{N_{d}}{\underset{n=1}{\prod }}\overset{K}{\underset{k=1}{\sum }}\theta_{dk}\phi_{k,w_{dn}} \end{array}$$

where *w* denotes the words in documents, θ_*dk*_ is the topic distribution for document *d*, ϕ_*k*,_*w*__*dn*__ is the word distribution for topic *k*, *D* is the total number of documents, and *N*_*d*_ is the number of words in document *d*.

(2) Non-negative Matrix Factorization (NMF). Decomposes the document-word matrix *V* into the product of a document-topic matrix *W* and a topic-word matrix *H*, with the number of topics set to *K* = 20. The objective is to minimize reconstruction error:

(16) $$\begin{array}{l} \underset{W,H}{\min}∥V-WH{∥}_{F}^{2} \end{array}$$

where $∥\cdot {∥}_{F}^{2}$ denotes the Frobenius norm.

(3) Top2Vec (35). A joint embedding and hierarchical clustering model that automatically infers the number of topics. It uses the Universal Sentence Encoder for document embedding and does not require predefining the number of topics (36).
(4) Original BERTopic Model. This BERT-based topic modeling method integrates BERT-derived embeddings, UMAP for dimensionality reduction (n_neighbors = 30, n_components = 5, metric = “cosine”), and HDBSCAN for clustering (min_cluster_size = 100, metric = “euclidean”).

#### 3.4.2 Performance metrics for individual modules

To assess the effectiveness of the three proposed modules, Popularity Deviation Regularizer (PDR), Dynamic Document Embedding Optimizer (DDEO), and Probabilistic Reassignment Matrix (PRM), we employed four evaluation metrics, each selected to align with the specific objective of its corresponding module.

(1) Lexical Diversity (LD). Used to measure the uniqueness of keywords within each topic. A higher LD indicates reduced redundancy among top words, reflecting the PDR module's ability to suppress generic terms and enhance semantic specificity. LD was computed using basic set operations and token counting functions from Python's built-in libraries and NumPy.
(2) Cosine Similarity (CS). Applied to PDR as well, CS quantifies the semantic proximity between document embeddings and their assigned topic centroids. A lower CS score after regularization suggests reduced embedding homogenization caused by frequent terms. The cosine similarity was calculated using the cosine_similarity function from scikit-learn.
(3) Silhouette Coefficient (SC). Used to evaluate DDEO's impact on clustering structure. SC measures the cohesion and separation of clusters, with values closer to one indicating better-defined topic boundaries in the reduced embedding space. SC was computed using silhouette_score from scikit-learn.
(4) Outlier Rate (OR). Used to evaluate PRM. OR reflects the proportion of tweets labeled as noise (i.e., not assigned to any topic) during clustering. A lower OR after applying PRM indicates improved document retention and topic coverage.

#### 3.4.3 Overall topic modeling evaluation metrics

To assess the effectiveness of the proposed topic modeling methods, we used the following standard evaluation metrics:

(1) Normalized Pointwise Mutual Information (NPMI). Evaluates semantic coherence by measuring the co-occurrence of top words within each topic. Higher values indicate stronger internal consistency. Computed using the Palmetto coherence library.
(2) Topic Diversity (TD). Measures the uniqueness of keywords across topics. A higher score suggests that different topics are well-separated and exhibit less keyword overlap. Calculated using custom Python scripts based on set operations.
(3) Perplexity. Reflects the model's ability to predict unseen data. Although more suited for probabilistic models (e.g., LDA), it was included here for comparative purposes and computed using Gensim's perplexity scoring method.

## 4 Results

### 4.1 Model comparison

#### 4.1.1 Validation of key innovations

To evaluate the contributions of the three proposed modules, namely Popularity Deviation Regularizer (PDR), Dynamic Document Embedding Optimizer (DDEO), and Probabilistic Reassignment Matrix (PRM), we conducted ablation experiments on each component within the BERTopic framework.

To evaluate the effectiveness of the three proposed modules, we used the following metrics aligned with each module's design objective: Lexical Diversity (LD) and Cosine Similarity (CS) for PDR, Silhouette Score (SC) for DDEO, and Outlier Rate (OR) for PRM. These metrics, respectively capture improvements in semantic richness, embedding structure, clustering quality, and document retention.

As shown in Table 4, in terms of lexical diversity, BERTopic_PDR achieved a score of 0.4643, significantly higher than the original BERTopic model (0.3381), indicating that PDR effectively suppressed high-frequency generic terms while enhancing the weight of domain-specific vocabulary, resulting in a more balanced lexical distribution across documents.

**Table 4:** Performance impact of PDR on BERTopic.

**Model**	**Evaluation metrics**	**Score**
BERTopic	Lexical diversity	0.3381
BERTopic_PDR		0.4643
BERTopic	Cosine similarity	0.7336
BERTopic_PDR		0.6195
Reduction ratio		0.1542
Boosting rate		1.7628

In terms of cosine similarity, BERTopic_PDR recorded a score of 0.6195, which is 0.1141 lower than the original model's 0.7336. This reduction suggests that PDR diminished the influence of redundant high-frequency words and improved inter-topic separability, thereby enhancing semantic clarity and overall model interpretability.

Furthermore, the reduction ratio for high-frequency terms reached 0.1542, and the boosting rate for domain-specific terms was 1.7628, further validating that PDR successfully reduced irrelevant lexical noise while amplifying topic-relevant terminology critical to the target domain.

**Figure 3:**
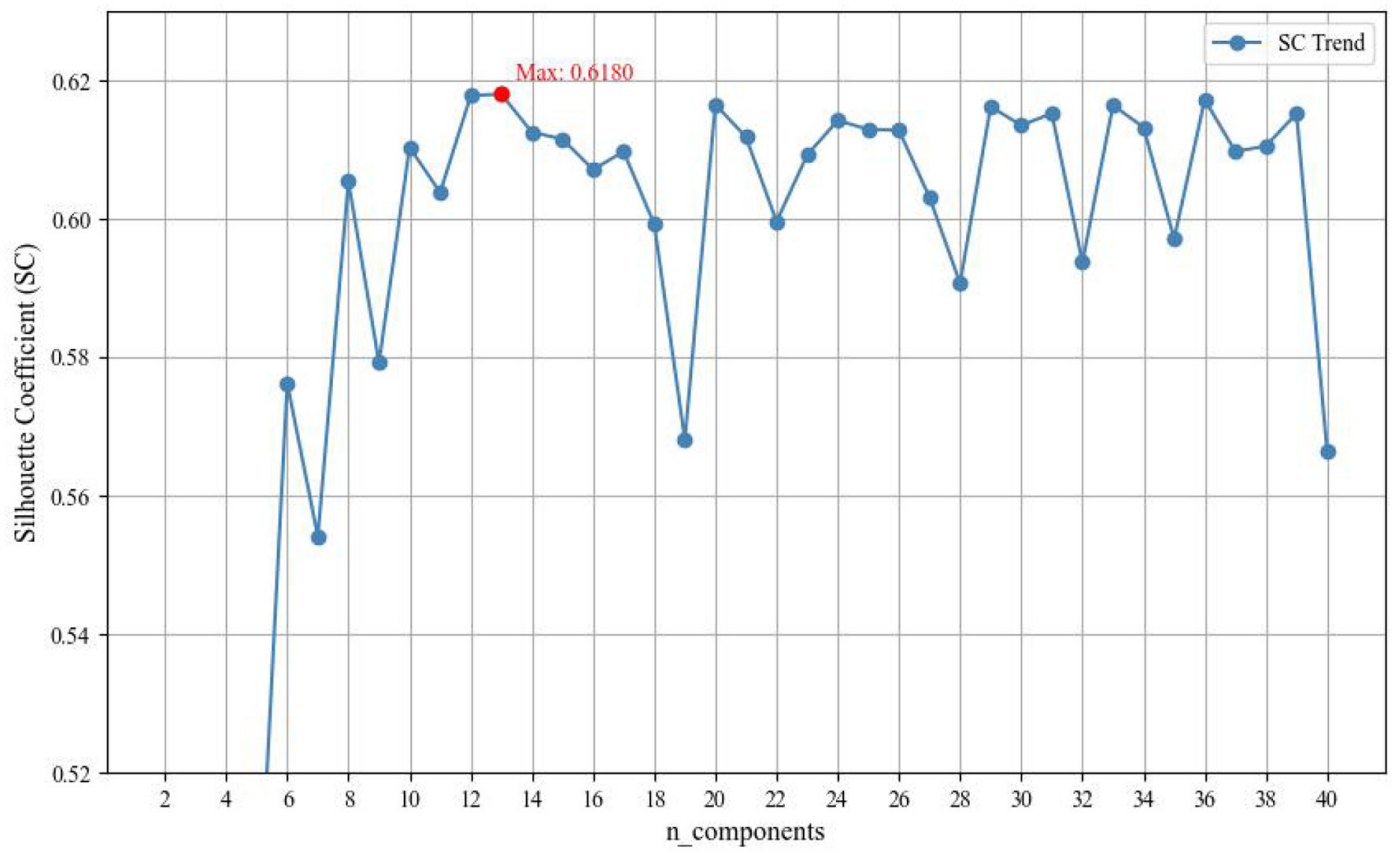
Optimization of UMAP dimensions in DDEO based on silhouette score.

Experimental results in Figure 3 demonstrate that the Dynamic Document Embedding Optimizer (DDEO) has a significant impact on topic modeling performance across different UMAP dimensions (n_components). When n_components = 2, the Silhouette Score (SC) was only −0.6309, indicating poor clustering performance due to excessively low dimensionality, which failed to effectively separate topics.

As the dimensionality increased from 2 to 40, the SC values initially rose, reflecting improved topic separability, and peaked at 0.6180 when n_components = 13. Beyond this point, performance leveled off or slightly declined, indicating that an embedding with 13 dimensions offered the best trade-off between topic cohesion and semantic richness.

However, further increasing the dimensionality led to a decline in performance, as the SC dropped to 0.6071, 0.5992, and 0.5681 at n_components = 16, 18, and 19, respectively. This suggests that overly high dimensionality may introduce noise and reduce topic clarity. Therefore, DDEO proves effective in improving topic coherence and distinctiveness by adaptively selecting the optimal embedding dimension within the BERTopic framework.

To determine an appropriate value for the min_cluster_size parameter in HDBSCAN, we conducted baseline experiments using the standard BERTopic framework under five candidate settings: 30, 50, 100, 200, and 300. As shown in Table 5, smaller values (e.g., 30 or 50) produced an excessive number of fragmented topics, many of which lacked semantic cohesion or interpretability. On the other hand, larger values (e.g., 200 or 300) led to fewer topics but a significantly higher Outlier Rate (OR), indicating many tweets were discarded during clustering. A value of 100 was selected as a balance point, yielding a manageable number of coherent topics with an acceptable OR.

**Table 5:** Effect of min_cluster_size on topic count and outlier rate.

**min_cluster_size**	**Number of topics**	**Outlier count**	**Outlier rate**
30	325	4,296	6.67%
50	175	9,021	14.01%
100	50	17,670	27.43%
200	36	24,883	39.63%
300	23	29,302	45.50%

In this study, given the large dataset size (a total of 64,407 tweets), the HDBSCAN clustering algorithm was configured with min_cluster_size = 100, meaning that a group of documents is considered a valid cluster only if at least 100 samples share similar embedding characteristics. Documents that fail to meet this condition are labeled as outliers, resulting in a relatively high Outlier Rate (OR).

Without applying the Probabilistic Reassignment Matrix (PRM), the OR reached 27.43% (see Table 6), indicating that a substantial portion of documents was discarded due to the strict hard clustering criteria of HDBSCAN. To address this, PRM targets only those documents initially labeled as outliers by HDBSCAN (i.e., assigned label −1) and reassigns them to the most semantically relevant topics based on embedding similarity to topic centroids. This mechanism not only preserves documents with potentially meaningful content that were previously discarded, but also enhances topic coverage and coherence, especially for weak or emerging themes that may be underrepresented in hard clustering.

**Table 6:** Outlier rate comparison between BERTopic and BERTopic_PRM.

**Model**	**OR**
BERTopic	17,670 (27.43%)
BERTopic_PRM	3,368 (5.23%)

Experimental results show that after applying PRM, the OR significantly decreased to 5.23%, corresponding to 3,368 outlier documents. This means that ~80% of the outliers were successfully reassigned to appropriate topic clusters. These findings confirm that PRM effectively reduces invalid outlier assignments and preserves the semantic integrity of the corpus. The adjustment not only lowers the outlier rate but also improves overall topic coherence and interpretability.

#### 4.1.2 Ablation study

To evaluate the individual contributions of the PDR, DDEO, and PRM modules, eight experiments were performed to assess the impact of each module on topic modeling performance. The purpose of these experiments was to isolate the effect of each module by evaluating various model configurations. The performance of each model variant was assessed using three key metrics: NPMI coherence, Topic Diversity (TD), and Perplexity. The experimental results are summarized in Table 7.

**Table 7:** Ablation experiment results.

**ID**	**Modules**			**Evaluation metrics**		
	**PDR**	**DDEO**	**PRM**	**NPMI**	**TD**	**Perplexity**
1	×	×	×	0.1882	0.9867	2.0580
2	√	×	×	0.2013	0.9917	2.0281
3	×	√	×	0.1627	0.9816	1.6084
4	×	×	√	0.1882	0.9867	2.0580
5	√	√	×	0.1531	0.9813	1.6698
6	√	×	√	0.2013	0.9917	2.0281
7	×	√	√	0.1627	0.9816	1.6084
8	√	√	√	0.2184	0.9935	1.7214

“ × ”: this policy is not used; “√”: this policy is used.

The Topic Diversity (TD) scores in this study were generally high, primarily due to the inherent independence among subdomains within adolescent health topics. Areas such as mental health, nutrition, screen use, school bullying, and addictive behaviors exhibit clear semantic separation, resulting in minimal keyword overlap. Consequently, the topic modeling process naturally produced well-differentiated and non-overlapping topics, contributing to high TD scores.

In Experiment 2, the PDR module significantly improved both NPMI (0.2013 vs. 0.1882) and TD (0.9917 vs. 0.9867), while slightly reducing Perplexity (2.0281 vs. 2.0580). These results suggest that PDR enhances topic coherence and separability by suppressing redundant high-frequency terms and reinforcing domain-specific keywords.

The DDEO module, tested in Experiment 3, primarily improved document embedding quality, leading to a substantial reduction in Perplexity (1.6084 vs. 2.0580). However, its effect on NPMI and TD was limited, and in some cases slightly negative, indicating that while DDEO improves model stability, it has minimal impact on keyword-level topic coherence.

The PRM module focuses on reducing invalid outlier classifications. In Experiment 4, using PRM alone resulted in metric values identical to the baseline, confirming that PRM does not directly affect NPMI or TD. However, in Experiment 7 (DDEO + PRM), Perplexity reached its lowest value (1.6084), suggesting that PRM, when supported by improved embeddings, further optimizes topic assignment. Similarly, in Experiment 6 (PDR + PRM), PRM preserved the performance gains of PDR in both NPMI and TD.

In Experiment 8, where PDR, DDEO, and PRM were combined, the model achieved the best overall performance: NPMI reached 0.2184, TD peaked at 0.9935, and Perplexity dropped to 1.7214. These results indicate that PRM plays a critical role in reducing outlier noise and, when integrated with PDR and DDEO, contributes to a more stable and higher-quality topic distribution.

#### 4.1.3 Comparative study

**Figure 4:**
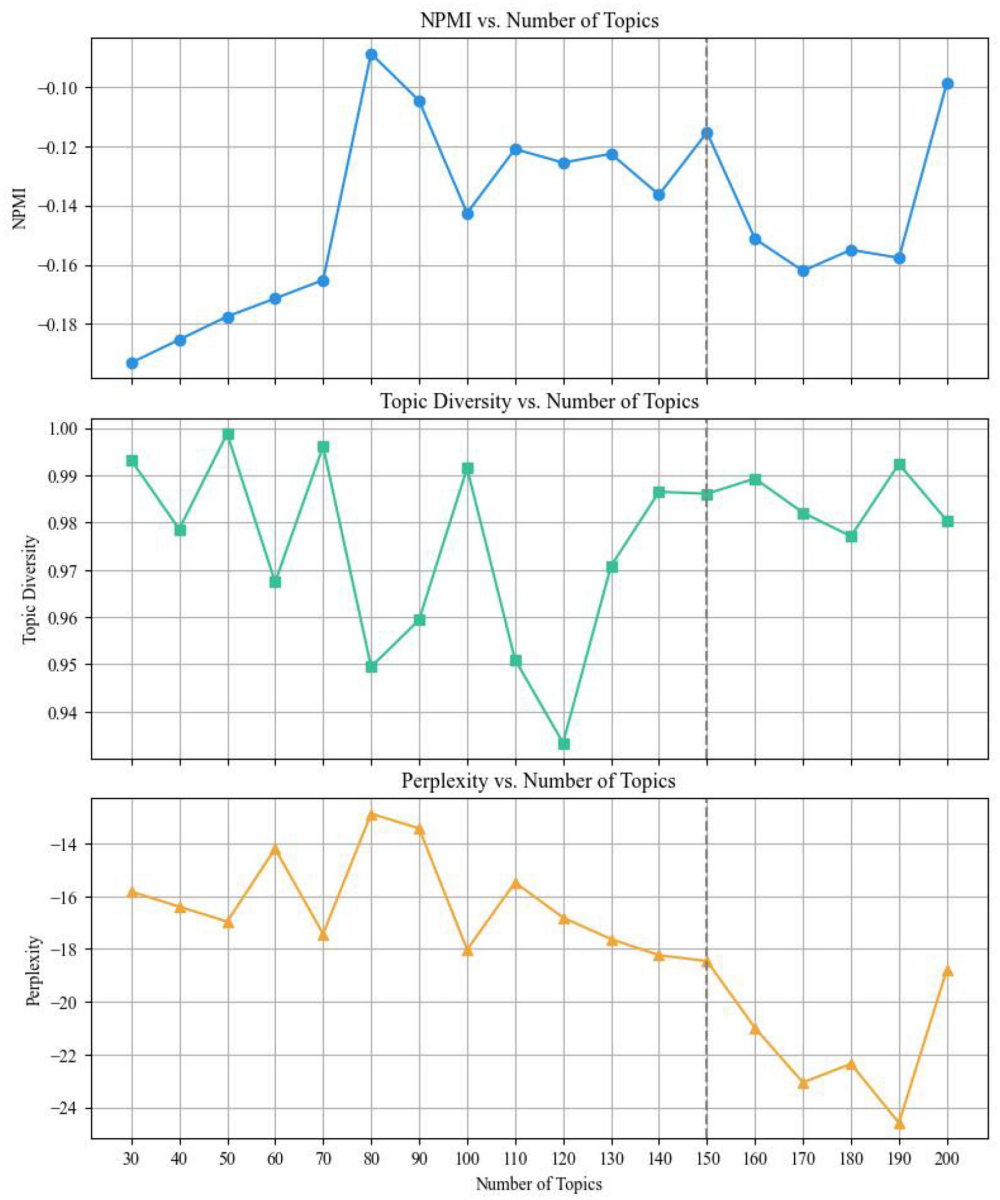
Evaluation metrics of LDA under varying topic numbers.

To ensure a fair and comprehensive comparison, we conducted additional experiments with LDA and NMF under varying topic numbers. The results are presented in Tables 8, 9. The evaluation results for LDA under varying topic numbers are presented in Figure 4.

**Table 8:** Performance of LDA under varying topic numbers.

**Number of topics**	**Evaluation metrics**		
	**NPMI**	**TD**	**Perplexity**
30	−0.1931	0.9932	−15.8234
40	−0.1853	0.9786	−16.3923
50	−0.1775	0.9988	−16.9605
60	−0.1714	0.9674	−14.1953
70	−0.1652	0.9961	−17.4301
80	−0.0888	0.9495	−12.8715
90	−0.1047	0.9594	−13.425
100	−0.1426	0.9915	−18.0219
110	−0.121	0.951	−15.4753
120	−0.1256	0.9334	−16.8076
130	−0.1226	0.9708	−17.6233
140	−0.1364	0.9865	−18.2355
150	−0.1154	0.9861	−18.4568
160	−0.1513	0.9893	−20.9934
170	−0.1621	0.9821	−23.0595
180	−0.155	0.9771	−22.3537
190	−0.1577	0.9924	−24.5947
200	−0.0987	0.9804	−18.7721

**Table 9:** Performance of NMF under varying topic numbers.

**Number of topics**	**Evaluation metrics**		
	**NPMI**	**TD**	**Perplexity**
30	0.1425	0.9732	2.1195
50	0.1691	0.9829	2.0003
70	0.1735	0.9784	1.9121
100	0.1668	0.9742	1.8547
150	0.1493	0.9654	1.7902
200	0.1315	0.9601	1.7453

For LDA, at *k* = 70, the NPMI reached −0.1652, topic diversity (TD) was at 0.9961, and perplexity was −17.4301. Although increasing *k* further (e.g., 150) continued to reduce perplexity slightly, NPMI began to plateau, and topic diversity fluctuated. These trends suggest that *k* = 70 provides the most suitable configuration, balancing semantic coherence, model fit, and topic diversity effectively without over-fragmentation. In contrast, NMF exhibited a peak in coherence (NPMI = 0.1735) at *k* = 70, with stable perplexity and acceptable topic diversity. Beyond this point, coherence began to decline, indicating that the model was generating overly fragmented topics.

Based on these observations, we selected *k* = 70 for LDA and *k* = 70 for NMF as the most appropriate configurations for our experiments.

In this experiment, we compared the topic modeling performance of LDA, NMF, Top2Vec, BERTopic, and the proposed BERTopic_Teen on the same dataset (results shown in Table 10), using NPMI, Topic Diversity, and Perplexity as evaluation metrics.

**Table 10:** Comparison experiments of different models.

**Model**	**Number of topics**	**Evaluation metrics**		
		**NPMI**	**TD**	**Perplexity**
LDA	70	−0.1652	0.9961	−17.4301
NMF	70	0.1735	0.9784	1.9121
Top2Vec	396	−0.2111	0.8745	1.1793
BERTopic	50	0.1882	0.9867	2.0580
BERTopic_Teen	55	0.2184	0.9935	1.7214

It is worth noting that the compared models differ in how the number of topics is determined. The BERT-based models (BERTopic and BERTopic_Teen) as well as Top2Vec adopt automatic topic number estimation. Specifically, Top2Vec generated 396 topics, BERTopic produced 50, and BERTopic_Teen, after additional optimization, produced 55 topics. In contrast, LDA and NMF require a predefined number of topics. To ensure a fair and rigorous comparison, we performed additional experiments by tuning the number of topics for both LDA and NMF across a range of values (30–200). Based on the evaluation metrics, we identified the optimal configurations as LDA with 70 topics and NMF with 70 topics.

In terms of performance, BERTopic_Teen achieved the best results on both NPMI (0.2184) and TD (0.9935), indicating an effective balance between topic coherence and diversity. LDA, at its optimal setting (70 topics), reached a balanced configuration with a NPMI of −0.1652, a high topic diversity (TD = 0.9961), and a relatively low perplexity (−17.4301). NMF, at 70 topics, achieved stronger coherence (NPMI = 0.1735) and moderate perplexity (1.9121), demonstrating balanced interpretability and coherence. In contrast, Top2Vec, which generated a large number of topics (396), had a lower TD (0.8745) and negative NPMI (−0.2111), indicating weaker coherence and more fragmented topic formation.

### 4.2 Summary of experimental results

This study compared five topic modeling methods, namely LDA, NMF, Top2Vec, BERTopic, and the proposed BERTopic_Teen, to evaluate their performance in analyzing adolescent health-related data from social media. Experimental results indicate that BERTopic_Teen outperforms all other models, validating the effectiveness of the proposed optimization strategies: PDR, DDEO, and PRM.

BERTopic_Teen achieved an NPMI score of 0.2184, representing a 16.0% improvement over the original BERTopic (0.1882), indicating enhanced semantic coherence among topic keywords. In comparison, LDA (70 topics) and Top2Vec (396 topics) yielded negative NPMI scores (−0.1652 and −0.2111, respectively), reflecting poor topic quality and a lack of meaningful lexical associations in the short-text environment of social media data.

The Topic Diversity (TD) score for BERTopic_Teen reached 0.9935, suggesting a well-balanced distribution of distinctive topics with minimal keyword redundancy. This is consistent with the diverse yet separable nature of adolescent health discussions, where subdomains such as mental health, lifestyle behaviors, and digital media are commonly discussed in isolation.

Regarding perplexity, BERTopic_Teen achieved a score of 1.7214, lower than the original BERTopic (2.0580) and NMF (1.9121 at 70 topics), indicating more stable and confident topic assignments. LDA (70 topics) achieved a well-balanced configuration with a relatively low perplexity (−17.4301) and high topic diversity (0.9961), suggesting effective topical separation. This configuration also exhibited a more favorable NPMI score of −0.1652 compared to higher topic settings, indicating better semantic coherence. Top2Vec achieved the lowest perplexity (1.1793), but generated 396 topics, which significantly reduced its TD (0.8745), indicating overly fragmented topic distributions that compromise interpretability. The behavior can be attributed to the core mechanism of Top2Vec, which detects topic vectors based on the clustering of document embeddings in a continuous semantic space. While this approach does not require the number of topics to be preset, it is highly sensitive to noise and lexical variation—common characteristics in short-form, user-generated texts such as tweets. Minor differences in spelling, grammar, or phrasing can result in semantically similar content being split into multiple clusters. Additionally, Top2Vec lacks post-processing procedures to consolidate redundant topics or penalize generic terms, further exacerbating topic overlap and reducing clarity.

To assess the computational feasibility of our proposed approach, we recorded the approximate runtime required for processing the full dataset of 64,407 tweets. All experiments were conducted on a workstation equipped with an NVIDIA RTX 3090 GPU and 128 GB of RAM. Using the complete BERTopic_Teen pipeline—including the Popularity Deviation Regularizer (PDR), Dynamic Document Embedding Optimizer (DDEO), and Probabilistic Reassignment Matrix (PRM)—the end-to-end process took ~2.5 h. This includes document embedding generation, dimensionality reduction via UMAP, HDBSCAN clustering, and post-processing steps. While the model is more computationally intensive than traditional methods such as LDA, its performance benefits and modular structure make it feasible for most academic or applied research settings.

## 5 Discussion

### 5.1 Topic overview

**Figure 5:**
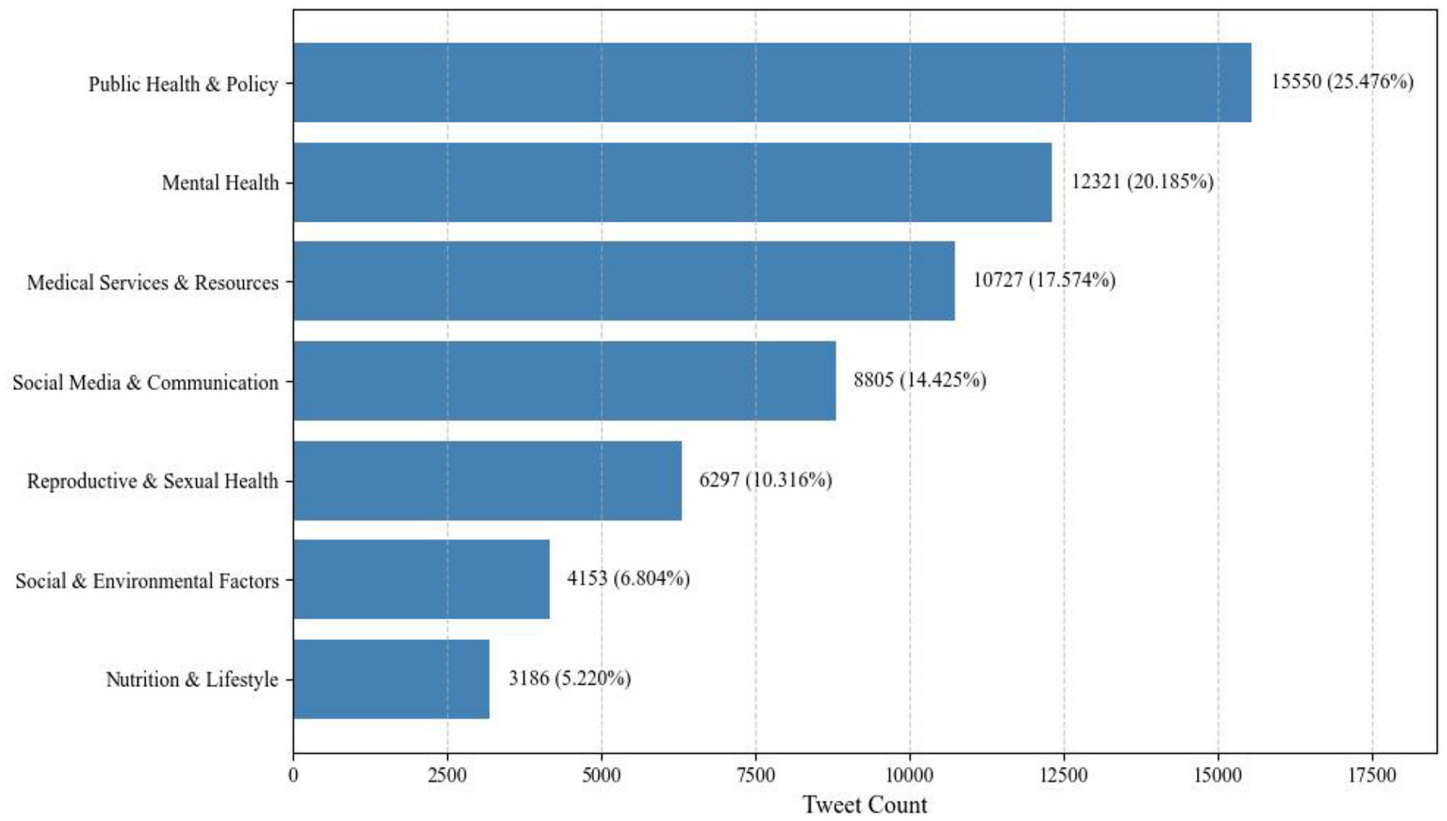
Tweet distribution across thematic dimensions.

A total of 61,039 adolescent health-related tweets were successfully classified, resulting in seven core thematic domains covering a wide range of issues, including mental health, substance use, and access to medical services. To improve interpretability, topic labels were manually assigned based on both the top-ranked keywords and representative tweets within each cluster. Two researchers with backgrounds in public health and computational social science independently proposed the labels, and any discrepancies were resolved through discussion until consensus was reached. The thematic distribution and corresponding subtopics are presented in Figure 5 and Table 11.

**Table 11:** Subject distribution situation.

**Dimensions**	**Specific topic**	**Quantity**
Public health and policy	Youth health week	571 (0.935%)
	Youth health policy	323 (0.529%)
	Youth health and awards	3,066 (5.023%)
	The lancet child health research	3,356 (5.498%)
	Youth health research	462 (0.757%)
	Youth health in East Africa	1,422 (2.330%)
	Indian youth health	1,995 (3.268%)
	Vaccination and immunization	2,231 (3.655%)
	Healthy day and public activities	703 (1.152%)
	Nigerian youth health	352 (0.577%)
	COVID-19 pandemic's impact on teenagers	1,069 (1.751%)
Mental health	Mental health of adolescents	4,545 (7.446%)
	Autism and related research	1,251 (2.050%)
	Prevention of suicide and self-harm	978 (1.602%)
	Anxiety and depression	816 (1.337%)
	Mental health institution	597 (0.978%)
	ADHD (attention-deficit/hyperactivity disorder)	496 (0.813%)
	Gender identity and mental health	390 (0.639%)
	Trauma and mental health	564 (0.924%)
	Attachment relationships and mental health	849 (1.391%)
	Resilience and mental health	765 (1.253%)
	Art and mental health	157 (0.257%)
	Marijuana use and adolescent mental health	1,251 (2.050%)
Medical services and resources	Medical services and funding	1,103 (1.807%)
	CAMHS (child and adolescent mental health services)	615 (1.008%)
	Nurse profession and mental health services	875 (1.434%)
	Medical waiting times and accessibility	376 (0.616%)
	Medical services and referrals	948 (1.553%)
	Adolescent psychiatry and mental health services	2,370 (3.883%)
	Youth health-related profession	1,154 (1.891%)
	Mental health services for adolescents	2,628 (4.305%)
	Challenges in CAMHS and support appeals	658 (1.078%)
Social media and communication	Internet link and social media spread	6,019 (9.861%)
	Podcast and mental health communication	820 (1.343%)
	Social media and mental health	1,242 (2.035%)
	Digital health and technology	724 (1.186%)
Reproductive and sexual health	Maternal and infant health and newborn care	1,731 (2.836%)
	Sexual health and reproductive health	350 (0.573%)
	Reproductive health and maternal and child health	422 (0.691%)
	AIDS and adolescent health	533 (0.873%)
	Menstrual hygiene and health	2,322 (3.804%)
	Transgender youth health	530 (0.868%)
	Adolescent abortion and the law	409 (0.670%)
Social and environmental factors	Social media use and behavior	398 (0.652%)
	Climate change and children's health	287 (0.470%)
	Youth violence and relationships	827 (1.355%)
	The impact of racism on teenagers	737 (1.207%)
	Substance abuse and addiction	365 (0.598%)
	Cyberbullying and school bullying	1,300 (2.130%)
	Youth social welfare and policy	239 (0.392%)
Nutrition and lifestyle	Dietary health and dietary disorders	1,098 (1.799%)
	Sleep health and psychological impact	749 (1.227%)
	Physical activity and health	640 (1.049%)
	Smoking and e-cigarettes	408 (0.668%)
	Fetal alcohol syndrome (FASD)	291 (0.477%)

**Figure 6:**
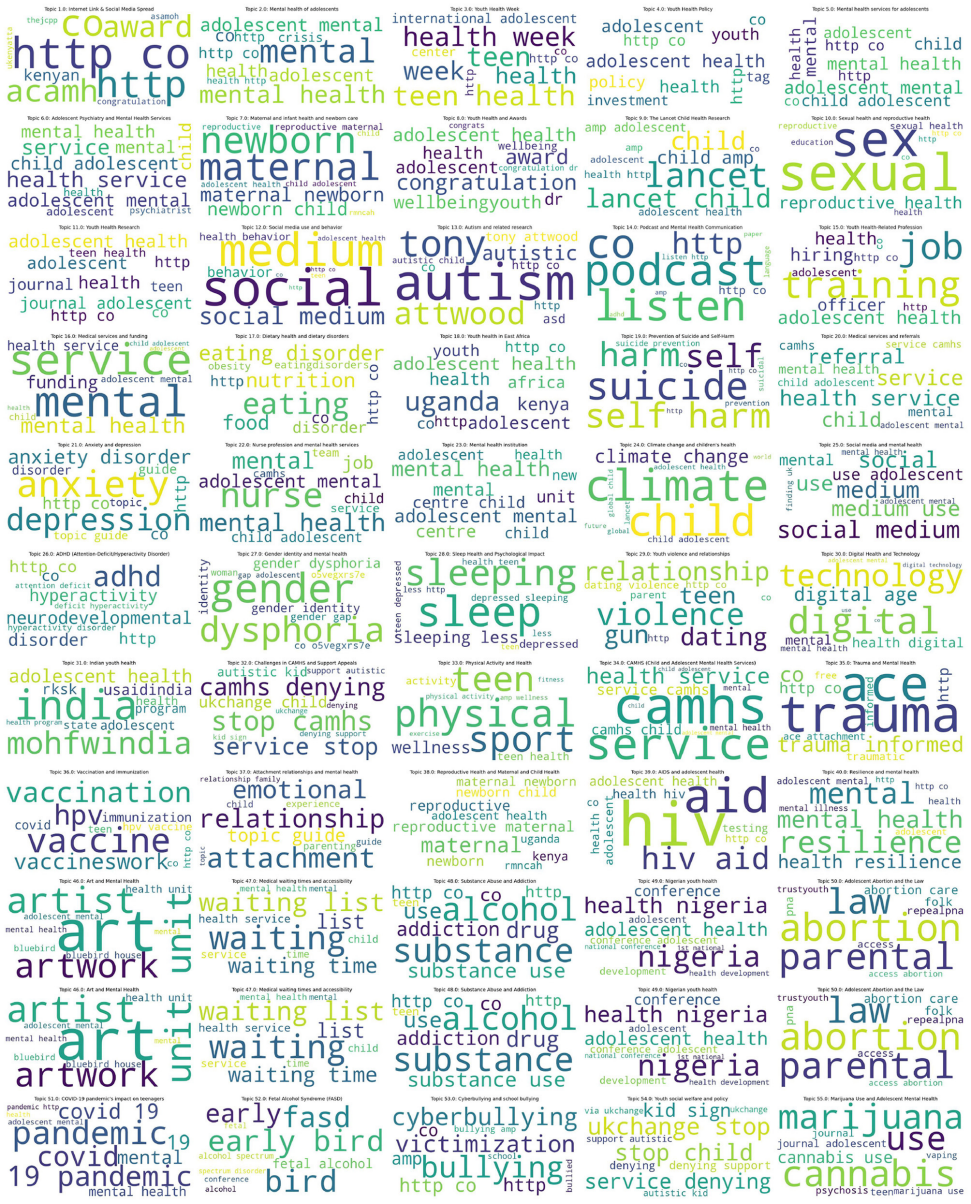
Word cloud visualizations of topics identified by the BERTopic_Teen model.

To further enhance interpretability, word clouds were generated for each of the identified topics based on their top-ranked keywords. Figure 6 displays examples of these word clouds, offering a visual summary of the semantic focus within each thematic domain.

Public Health and Policy (25.476%) emerged as the most prominent domain. Its visibility is largely driven by policy implementation and region-specific health initiatives. For instance, the topic “Youth Health Week” (5.498%) triggered a peak daily tweet volume of over 1,200, fueled by online–offline integrated activities such as health screenings and vaccination campaigns. Regional topics like “Indian youth health” (1.751%) centered on sharing practical experience in malaria prevention, highlighting the health assistance needs of developing countries. Meanwhile, “The Lancet Child Health Research” (3.268%) demonstrated viral spread among parent communities.

Mental Health (20.185%) ranked second, presenting a mix of traditional and emerging issues. While “Mental health of adolescents” (7.446%) remains a central topic, discussions have expanded from academic stress to digital generational conflicts. Notably, “Autism and related research” (2.050%) surpassed “ADHD” (1.337%) in volume for the first time. Many tweets advocated for educational reform, such as a viral U.S. proposal to mandate autism counselors in public schools, which was retweeted over 320,000 times, reflecting an evolving societal awareness of neurodiversity.

Medical Services and Resources (17.574%) revealed systemic tensions in healthcare delivery. Tweets under “Mental health services for adolescents” (4.305%) reported that 81% of cases faced wait times exceeding 6 months. Discussions around “Adolescent Psychiatry and Mental Health Services” (3.883%) focused on the monopolization of private mental health resources. Meanwhile, “Nurse profession and mental health services” (1.434%) highlighted the positive role of frontline nurses in developing countries, with 86% of tweets praising their contributions to school-based mental health screening.

### 5.2 Key drivers of attention differentiation

Public health policies played a pivotal role in driving attention to youth health topics on social media. Policy-related campaigns, such as “Youth Health Policy” and “Youth Health Week,” achieved wide visibility through digital dissemination, catalyzing broad social engagement. These campaigns often integrated online health screenings and vaccination efforts, exemplifying action-oriented policy design with high interaction and diffusion rates.

In the mental health domain, topics such as suicide prevention and art therapy showed distinct temporal spikes. Suicide prevention tweets surged during examination seasons, reflecting academic stress as a critical trigger of youth mental distress. Art therapy emerged as an innovative intervention strategy; for instance, the #MentalHealthArtChallenge attracted substantial youth participation. This interactive campaign bridged online discourse with offline practices, demonstrating the potential of digital engagement in promoting mental health literacy.

Although “Adolescent Psychiatry and Mental Health Services” (3.883%) was widely discussed, youth still face major barriers in accessing professional care. Many tweets referenced prolonged wait times, which not only delay treatment but risk worsening conditions. Furthermore, tweets under “Mental health services for adolescents” (4.305%) emphasized increasing demand amid insufficient supply, particularly within CAMHS (Child and Adolescent Mental Health Services) and referral mechanisms, highlighting critical issues of coverage and timeliness.

Social media demonstrated a dual effect in health communication. While social media links (9.861%) facilitated rapid information dissemination, they also contributed to content fragmentation and misinformation, for example, erroneous claims about e-cigarette safety. In contrast, long-form content such as podcasts enabled deep vertical discussions. Topics like school violence gained traction through such formats, which boosted outreach for non-profit mental health organizations. This suggests that high-quality content holds irreplaceable value in advancing targeted public engagement and intervention.

### 5.3 Study limitations

Despite its strong performance, BERTopic_Teen has certain limitations. First, the model was trained and fine-tuned specifically for adolescent health data. While this ensures domain-specific performance, it may constrain the model's generalizability to other domains or broader public health applications. Second, due to its

reliance on deep learning-based embeddings, the computational cost is relatively high, limiting its scalability for real-time analysis. Third, perplexity may be less informative for short-text data, as it does not always reflect topic coherence, underscoring the need for complementary metrics such as NPMI and Topic Diversity.

## 6 Conclusion and future work

This study conducted a comparative evaluation of five topic modeling methods, namely LDA, NMF, Top2Vec, BERTopic, and the proposed BERTopic_Teen, on adolescent health-related social media data. To enhance model performance, we introduced three optimization strategies: PDR to mitigate the influence of high-frequency terms, DDEO to adaptively select UMAP dimensions, and PRM to reduce invalid outlier classifications. Experimental results show that BERTopic_Teen outperformed all baselines in terms of NPMI, Topic Diversity, and Perplexity, demonstrating improved accuracy in identifying health-related topics and enhanced modeling stability through effective outlier reassignment.

Future work could explore computationally efficient alternatives, such as lightweight embedding models or distributed computing frameworks. Additionally, integrating complementary techniques, such as sentiment analysis and causal inference, may help uncover the evolution and underlying drivers of youth health topics, further enhancing the real-world applicability of the modeling results.

## Data Availability

The original contributions presented in the study are included in the article/supplementary material, further inquiries can be directed to the corresponding authors.
